# Invasive Lionfish Drive Atlantic Coral Reef Fish Declines

**DOI:** 10.1371/journal.pone.0032596

**Published:** 2012-03-07

**Authors:** Stephanie J. Green, John L. Akins, Aleksandra Maljković, Isabelle M. Côté

**Affiliations:** 1 Department of Biological Sciences, Simon Fraser University, Burnaby, British Columbia, Canada; 2 Reef Environmental Education Foundation, Key Largo, Florida, United States of America; University of Canterbury, New Zealand

## Abstract

Indo-Pacific lionfish (*Pterois volitans* and *P. miles*) have spread swiftly across the Western Atlantic, producing a marine predator invasion of unparalleled speed and magnitude. There is growing concern that lionfish will affect the structure and function of invaded marine ecosystems, however detrimental impacts on natural communities have yet to be measured. Here we document the response of native fish communities to predation by lionfish populations on nine coral reefs off New Providence Island, Bahamas. We assessed lionfish diet through stomach contents analysis, and quantified changes in fish biomass through visual surveys of lionfish and native fishes at the sites over time. Lionfish abundance increased rapidly between 2004 and 2010, by which time lionfish comprised nearly 40% of the total predator biomass in the system. The increase in lionfish abundance coincided with a 65% decline in the biomass of the lionfish's 42 Atlantic prey fishes in just two years. Without prompt action to control increasing lionfish populations, similar effects across the region may have long-term negative implications for the structure of Atlantic marine communities, as well as the societies and economies that depend on them.

## Introduction

The successful invasion of a marine ecosystem by vertebrate predators is exceedingly rare [1]. Nevertheless, one such invasion is currently unfolding. Indo-Pacific lionfish (*Pterois volitans* and *P. miles*) have spread rapidly across the Western Atlantic, Caribbean and Gulf of Mexico, producing a marine predator invasion of unparalleled speed and magnitude. Lionfish were first reported off the southeast coast of Florida in the 1980s and have since become established to varying extents across the entire Caribbean region via larval dispersal in ocean currents [2]. These ambush predators consume a wide variety of native fish and invertebrate species at high rates, and are well defended from predation by venomous fin spines [3], [4].

There is growing concern, largely based on the results of small-scale experiments [5], that lionfish will affect the structure and function of invaded marine ecosystems (e.g. [6], [7]) but detrimental impacts on natural communities have yet to be measured. To determine whether predation by lionfish is having negative effects on native reef fish communities, we studied nine sites along a 15 km stretch of continuous reef off the southwest coast of New Providence Island, Bahamas (24°59.072 N, 77°32.207 W), where lionfish were first sighted in 2004. We conducted visual transect surveys of both native fish and lionfish, and identified lionfish prey through stomach contents analysis of 567 lionfish collected from the study reefs in 2008 and 2010. Standardized roving diver surveys conducted at the sites each year since 2004 were used to assess changes in lionfish abundance over time within the study area.

## Results and Discussion

**Figure 1:**
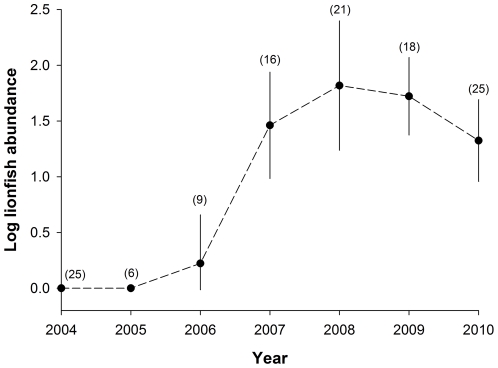
The abundance of Indo-Pacific lionfish (*Pterois volitans* and *P. miles*) on coral reefs off southwest New Providence, Bahamas. Abundance is the number of lionfish sighted during each roving survey, recorded in log_10_ scale. Points represent log-scale means, bounded by 95% confidence intervals. The yearly number of surveys is given in parentheses.

**Figure 2:**
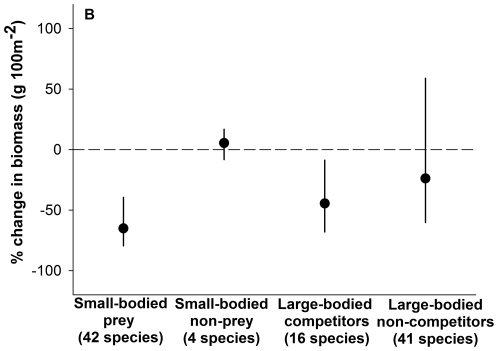
The percent change in biomass of native fishes between 2008 and 2010 on New Providence, Bahamas coral reef sites. Points represent medians, bounded by parametric bootstrapped 95% confidence intervals. The dashed line indicates no change in biomass.

Lionfish abundance increased swiftly between 2004 and 2010 off southwest New Providence, Bahamas (Figure 1). Between 2008 and 2010, abundant lionfish populations coincided with rapid declines in native fishes. During this period lionfish increased from 23% to nearly 40% of the total biomass of predators residing in the study area, which included 16 ecologically-similar native fishes, in terms of body size and diet [8], [9]. Ninety percent of the prey consumed by lionfish were small-bodied reef fishes from 42 species (Table S1). Between 2008 and 2010, the combined biomass of these 42 species declined by 65%, on average, across the study reefs (Figure 2; linear mixed-effects model (LMM); *P*<0.001, *t* = 4.5, *df* = 105). Since lionfish were already abundant within the study area in the year prior to our observations (Figure 1), the cumulative decline in prey fish biomass since lionfish first colonized the area undoubtedly exceeds what we observed between 2008 and 2010.

Aside from predation by lionfish, at least three alternative factors could cause such a rapid decline in the abundance of so many species: recruitment failure, increased predation by native species, or disease. Wholesale recruitment failure, owing to unfavourable oceanographic conditions for the pelagic larvae of reef fish, is unlikely to be a factor in the decline of lionfish prey, since the biomass of several species of small-bodied gobies (*Elacatinus* spp.; Table S1), which also have pelagic larvae but have never been recorded in diet of lionfish [3]–[5], [10] and may contain a chemical defense against predation [11], remained stable over the two-year period (Figure 2; LMM; *P* = 0.45, *t* = 0.78, *df* = 105). The decline in prey species was also not caused by an increase in native predators, as the biomass of the 16 ‘lionfish-analogous’ species also declined by 44% (Figure 2; LMM; *P* = 0.02, *t* = 2.1, *df* = 55), a change likely attributable to fishing pressure and/or competition with lionfish. By contrast, the biomass of non-predatory but large-bodied fishes, which were not vulnerable to lionfish predation (because they were already too large to be lionfish prey in 2008) or competition over this period but many of which are exploited to some degree, remained unchanged (Table S1; Figure 2; LMM; *P* = 0.13, *t* = 1.54, *df* = 55). Finally, no fish disease epidemic was reported during the study period, leaving lionfish predation as the most likely cause of the changes in prey fish abundance documented here.

Without prompt action, increasing lionfish populations are likely to have similar impacts on prey fish biomass across the region. The impacts of lionfish may not be limited to small-bodied prey species. In time, the abundance of large-bodied fishes which are consumed as juveniles by lionfish may be also be affected; these prey species fulfill important functional roles on coral reefs (Table S1). Given the broad geographic extent of the invasion, complete eradication of lionfish from the Atlantic appears unlikely [12]. However, lionfish control programs, which are being initiated across the Caribbean, may successfully mitigate the effects of lionfish at local scales within high-priority areas, such as Marine Protected Areas and fish nursery habitats [13]. In the absence of effective local action, the effects of the lionfish invasion may have long-term implications for the structure of Atlantic marine communities, as well as the societies and economies that depend on them.

## Materials and Methods

Our study took place at nine locations, each separated by at least 1 km, along a continuous stretch of coral reef bordering the Tongue of the Ocean trench off southwest New Providence, Bahamas. We estimated the size (total length (TL) to the nearest 1 cm) and density of all small-bodied and cryptic fishes (i.e. <15 cm TL) during detailed searches of 6–12 30 m×2 m (length×width) transects at each site in summers of 2008 and 2010. Size and density of larger-bodied (i.e. >15 cm TL) fishes were assessed on 2–6 30 m×4 m transects during the same periods. All transects were laid parallel to the reef crest at depths between 10–20 m at each site. Fish lengths (cm) were converted to body mass (g) using published species-specific allometric scaling constants obtained from FishBase [9] and verified in the primary literature. When species-specific constants were not available, we used allometric constants for closely related species with a similar body shape.

To test whether fish biomass (g 100 m^−2^) had changed significantly between 2008 and 2010, we created linear mixed-effects models, comparing the biomass of fish between years (fixed effect), while nesting transects within sites (random effects) [14]. To calculate the median percent change in fish biomass between 2008 and 2010 across the study system and 95% confidence intervals which incorporate variation among transects within sites, we first specified log-normal distributions for fish biomass at each site in 2008 and 2010. The mean and standard deviation of each distribution was calculated from transect data at each location. We next calculated the percent change in biomass between 2008 and 2010 for each site. To incorporate variation in our estimates of percent change, we conducted 1000 iterations of the calculation, using Monte Carlo simulation to draw from the distributions of biomass for each site [15]. We then calculated the average system-wide change in biomass from the medians of the site-specific percent-change distributions. We performed 500 replicates of this latter procedure to generate a distribution of values for system-wide percent change in biomass. Figure 2 displays the median of this bootstrapped distribution, with the 2.5 and 97.5 percentiles as our confidence limits. Between 2004 and 2010, lionfish abundance was recorded during roving diver surveys at the study sites by trained observers on SCUBA as part of the Reef Environmental Education Foundation (REEF) fish survey project [16]. Each survey consisted of a 30–60 min roving search of the site, during which the observer recording all species observed (including lionfish) as well as the categorical abundance of each species on a four-point log_10_ scale [single (1), few (2–10), many (11–100), and abundant (>100)] [17]. Data were entered into REEF's on-line data base at www.reef.org, where they passed through both an automated electronic and program manager review to ensure accuracy and completeness. Automated electronic checks included comparison to existing data from the site and flagging of species identification or abundance parameters outside existing data boundaries. All potential species/abundance outliers were confirmed with the observer by the program manager before processing was completed. Surveys which did not pass this quality assurance process were not included in the database.

Prey species for lionfish were determined from the stomach contents of the 567 lionfish specimens collected from the study sites between 2008 and 2010. Lionfish were collected using hand nets and euthanized at the surface in a clove oil and sea water solution. Stomach contents were then extracted and identified visually to the lowest taxonomic resolution possible. The collection and handling of all lionfish specimens for this study was approved by the Simon Fraser University Animal Care Committee and met Canadian Council on Animal Care animal usage guidelines and policies (permit 947B-09).

## Supporting Information

Table S1 **Species and size classes included in each of the four categories considered in the analysis of biomass change between 2008 and 2010 on nine coral reefs off southwest New Providence, Bahamas.** Fishes of <13 cm were deemed to be potential prey based on the maximum prey size observed in lionfish stomachs at these sites. Functional group was determined from diet composition [8] and trophic group [9]. *Species which are commercially exploited in the Bahamas.
(DOCX)
