# Supplementary material for: Invasive Lionfish Drive Atlantic Coral Reef Fish Declines
Source: PLoS One. 2012 Mar 7;7(3):e32596. doi: 10.1371/journal.pone.0032596 (PMC3296711; doi:10.1371/journal.pone.0032596)
Supplement: Table S1 — Species and size classes included in each of the four categories considered in the analysis of biomass change between 2008 and 2010 on nine coral reefs off southwest New Providence, Bahamas. Fishes of <13 cm were deemed to be potential prey based on the maximum prey size observed in lionfish stomachs at these sites. Functional group was determined from diet composition [8] and trophic group [9]. *Species which are commercially exploited in the Bahamas. (DOCX) [file pone.0032596.s001.docx]

**Table S1.** **Species and size classes included in each of the four categories considered in the analysis of biomass change between 2008 and 2010 on nine coral reefs off southwest New Providence, Bahamas.** Fishes of < 13 cm were deemed to be potential prey based on the maximum prey size observed in lionfish stomachs at these sites. Functional group was determined from diet composition [8] and trophic group [9]. *Species which are commercially exploited in the Bahamas.

| **Category** | **Family** | **Species** | **Functional group** |
| --- | --- | --- | --- |
| Large-bodied predatory fishes ecologically similar to lionfish based on diet and body size (Figure 2: “Large-bodied competitors”). Only individuals >25cm TL considered in this analysis. | Aulostomidae | *Aulostomus maculatus* | piscivore |
|  | Lutjanidae | *Lutjanus analis** | carnivore |
|  | Lutjanidae | *Lutjanus apodus** | carnivore |
|  | Lutjanidae | *Lutjanus cyanopterus** | carnivore |
|  | Lutjanidae | *Lutjanus griseus** | carnivore |
|  | Lutjanidae | *Lutjanus jocu** | carnivore |
|  | Lutjanidae | *Lutjanus mahogoni** | carnivore |
|  | Scorpaenidae | *Scorpaena plumieri* | piscivore |
|  | Serranidae | *Cephalopholis fulva** | piscivore |
|  | Serranidae | *Epinephelus adscensionis** | piscivore |
|  | Serranidae | *Epinephelus guttatus** | piscivore |
|  | Serranidae | *Epinephelus striatus** | piscivore |
|  | Serranidae | *Mycteroperca bonaci** | piscivore |
|  | Serranidae | *Mycteroperca interstitialis** | piscivore |
|  | Serranidae | *Mycteroperca tigris** | piscivore |
|  | Serranidae | *Mycteroperca venenosa** | piscivore |
| Large-bodied non-predatory fishes (Figure 2: “Large-bodied non-competitors”). Only individuals >25cm TL considered in this analysis. | Acanthuridae | *Acanthurus bahianus* | herbivore |
|  | Acanthuridae | *Acanthurus chirurgus* | herbivore |
|  | Balistidae | *Canthidermis sufflamen* | invertivore |
|  | Echeneidae | *Echeneis naucrates* | omnivore |
|  | Ephippidae | *Chaetodipterus faber* | omnivore |
|  | Haemulidae | *Anisotremus surinamensis** | invertivore |
|  | Haemulidae | *Anisotremus virginicus** | invertivore |
|  | Haemulidae | *Haemulon album** | invertivore |
|  | Haemulidae | *Haemulon carbonarium** | invertivore |
|  | Haemulidae | *Haemulon flavolineatum** | invertivore |
|  | Haemulidae | *Haemulon parra** | invertivore |
|  | Haemulidae | *Haemulon plumierii** | invertivore |
|  | Haemulidae | *Haemulon sciurus** | invertivore |
|  | Holocentridae | *Holocentrus adscensionis* | invertivore |
|  | Holocentridae | *Holocentrus rufus* | invertivore |
|  | Holocentridae | *Myripristis jacobus* | invertivore |
|  | Holocentridae | *Neoniphon marianus* | invertivore |
|  | Holocentridae | *Sargocentron vexillarium* | invertivore |
|  | Labridae | *Bodianus rufus* | invertivore |
|  | Labridae | *Halichoeres garnoti* | invertivore |
|  | Lutjanidae | *Ocyurus chrysurus** | planktivore |
|  | Monacanthidae | *Aluterus schoepfii* | herbivore |
|  | Monacanthidae | *Aluterus scriptus* | omnivore |
|  | Mullidae | *Mulloidichthys martinicus** | invertivore |
|  | Mullidae | *Pseudupeneus maculatus** | invertivore |
|  | Ostraciidae | *Lactophrys triqueter* | invertivore |
|  | Pomacanthidae | *Centropyge argi* | herbivore |
|  | Pomacanthidae | *Holacanthus ciliaris* | invertivore |
|  | Pomacanthidae | *Pomacanthus arcuatus* | omnivore |
|  | Pomacanthidae | *Pomacanthus paru* | omnivore |
|  | Scaridae | *Scarus coeruleus* | herbivore |
|  | Scaridae | *Scarus iserti* | herbivore |
|  | Scaridae | *Scarus taeniopterus* | herbivore |
|  | Scaridae | *Scarus vetula* | herbivore |
|  | Scaridae | *Sparisoma aurofrenatum* | herbivore |
|  | Scaridae | *Sparisoma rubripinne* | herbivore |
|  | Scaridae | *Sparisoma viride* | herbivore |
|  | Sparidae | *Calamus bajonado** | invertivore |
|  | Sparidae | *Calamus calamus** | invertivore |
| Small-bodied lionfish prey species identified from stomach contents (Figure 2: “Small-bodied prey”). Only individuals <13cm TL considered in the analysis. | Apogonidae | *Apogon planifrons* | invertivore |
|  | Apogonidae | *Apogon townsendi* | invertivore |
|  | Apogonidae | *Phaeoptyx pigmentaria* | invertivore |
|  | Atherinidae | *Atherinomorus sp.* | planktivore |
|  | Aulostomidae | *Aulostomus maculatus* | carnivore |
|  | Chaenopsidae | *Acanthemblemaria aspera* | planktivore |
|  | Chaenopsidae | *Lucayablennius zingaro* | planktivore |
|  | Gobiidae | *Coryphopterus bol* | omnivore |
|  | Gobiidae | *Coryphopterus eidolon* | omnivore |
|  | Gobiidae | *Coryphopterus glaucofraenum* | omnivore |
|  | Gobiidae | *Coryphopterus hyalinus* | planktivore |
|  | Gobiidae | *Coryphopterus personatus* | planktivore |
|  | Gobiidae | *Gnatholepis thompsoni* | omnivore |
|  | Gobiidae | *Lythrypnus spilus* | invertivore |
|  | Gobiidae | *Priolepis hipoliti* | invertivore |
|  | Grammatidae | *Gramma loreto* | invertivore |
|  | Holocentridae | *Sargocentron coruscum* | invertivore |
|  | Inermiidae | *Inermia vittata* | planktivore |
|  | Labridae | *Bodianus rufus* | invertivore |
|  | Labridae | *Clepticus parrae* | planktivore |
|  | Labridae | *Halichoeres bivittatus* | invertivore |
|  | Labridae | *Halichoeres garnoti* | invertivore |
|  | Labridae | *Halichoeres maculipinna* | invertivore |
|  | Labridae | *Thalassoma bifasciatum* | planktivore |
|  | Labrisomidae | *Labrisomus haitiensis* | invertivore |
|  | Labrisomidae | *Malacoctenus boehlkei* | invertivore |
|  | Monacanthidae | *Monacanthus tuckeri* | omnivore |
|  | Mullidae | *Pseudupeneus maculatus* | invertivore |
|  | Pomacentridae | *Chromis cyanea* | planktivore |
|  | Pomacentridae | *Chromis multilineata* | planktivore |
|  | Pomacentridae | *Stegastes partitus* | herbivore |
|  | Pomacentridae | *Stegastes variabilis* | herbivore |
|  | Scaridae | *Sparisoma aurofrenatum* | herbivore |
|  | Serranidae | *Cephalopholis cruentata* | carnivore |
|  | Serranidae | *Epinephelus striatus* | carnivore |
|  | Serranidae | *Hypoplectrus spp.* | carnivore |
|  | Serranidae | *Liopropoma rubre* | carnivore |
|  | Serranidae | *Serranus tabacarius* | carnivore |
|  | Serranidae | *Serranus tigrinus* | carnivore |
|  | Synodontidae | *Synodus intermedius* | piscivore |
|  | Synodontidae | *Synodus saurus* | piscivore |
|  | Synodontidae | *Synodus synodus* | piscivore |
| Small-bodied non-prey species (Figure 2: “Small-bodied non-prey”). All individuals <13cm TL. | Gobiidae | *Elacatinus chancei* | invertivore |
|  | Gobiidae | *Elacatinus evelynae* | invertivore |
|  | Gobiidae | *Elacatinus genie* | invertivore |
|  | Gobiidae | *Elacatinus horsti* | invertivore |
